# Changes in ankle joint alignment after proximal fibular osteotomy

**DOI:** 10.1371/journal.pone.0214002

**Published:** 2019-03-22

**Authors:** Jialiang Guo, Li Zhang, Di Qin, Wei Chen, Weichong Dong, Zhiyong Hou, Yingze Zhang

**Affiliations:** 1 Department of Orthopaedic Surgery, the Third Hospital of Hebei Medical University, Shijiazhuang, P R China; 2 Key Laboratory of Orthopaedic Biomechanics of Hebei Province, Shijiazhuang, P R China; 3 Orthopaedic Research Institution of Hebei Province, Hebei, P R China; 4 Department of Pharmacy, the Second Hospital of Hebei Medical University, Shijiazhuang, P R China; 5 Chinese Academy of Engineering, Beijing, P.R. China; Kanazawa University, JAPAN

## Abstract

The purpose of this study was to assess the effect of proximal fibular osteotomy (PFO) on ankle joint. 49 patients or 53 lower limbs were included and followed-up with a minimum of one year in the study prospectively. Patients were evaluated radiographically and clinically. The questionnaires of the American Knee Society Score (KSS), the Ankle-Hindfoot Scale of the American Orthopedic Foot and Ankle Society (AOFAS), Visual Analogue Scale/Score (VAS) were used to assess the patients clinically. Radiographic evaluations were measured by the hip-knee-ankle angle (HKA), the femoro-tibial angle (FTA), tibial inclination (TI), distal tibial articural surface (TAS), talar tilt (TT), length of fibular (FL), and hind foot alignment such as hindfoot alignment view angle (HAVA), hindfoot alignment ratio (HAR), and hindfoot moment arm (HMA). Of the 53 subjects, no significant differences were exhibited in AOFAS, VAS scores and FL in ankle joint, but a significant differences were demonstrated in KSS score, HKA, FTA, TI, TT, HAVA, HAR and HMA after PFO. Due to the structural improvements of ankle joint, PFO not only improves joint function but also the alignment of ankle joint radiographically, and is still recommended as a safe surgery in treating medial compartment osteoarthritis of knee.

## Introduction

Knee osteoarthritis (KOA) is a chronic, progressive, degenerative disease especially occurred commonly in older individuals. Knee varus deformities, characterized with bearing axis shifting towards the medial side and a narrowed medial joint space, are commonly observed in patients with KOA and affect 74% of patients [1]. Despite the fact that two joints sustain similar loads in same patient, osteoarthritic ankles are relatively rare [2–4]. However, osteoarthritis of ipsilateral ankle are encountered when lower limb deformity exists, and the pain or discomfort induced should be paid on special attentions [4].

High tibial osteotomy (HTO) is an effective surgical method for the treatment of medial compartmental osteoarthritis in active and young adult. The excessive mechanical overload on medial compartment is reduced and re-distributed after HTO. It delays the time for patients to undergo total knee arthroplasty (TKA) by slowing destructive changes on medial compartment. However, post-operative complications (increased risk of nonunion, surgical wound infection and so on), slow rates of pain mitigation and high medical cost encourage us to find other less invasive methods. In recent study, after removal of the proximal section of fibula, patients with medial compartment KOA show greater improvement in lower extremity alignment, knee range of motion, and local pain mitigation [1]. Therefore, proximal osteotomy fibula (PFO) is considered as an alternative, affordable, novel surgical procedure in developing countries that are still constrained by funding and advanced instrumentations.

However, the thing easily being neglected is that correction of lower extremity alignment with PFO leads inevitably to changes in the alignment of ankle joint. Patients suffering from osteoarthritis of knee and ipsilateral ankle are sometimes observed especially in severe varus or valgus deformity ones. The effects of PFO on knee have been well documented, but the impact on ankle joint is still unclear [1,5]. The aim of this present research is to evaluate the effect of PFO on ankle joint radiographically and clinically.

## Materials and methods

### Demographic data of patients

From March 2015 to May 2016, a prospective study of 54 patients diagnosed as medial compartmental OA with varus deformities of the knee were treated and followed up prospectively using a picture archiving and communication system workstation (PACS) from one center. The study was conducted in December 2017, all methods were performed in accordance with the relevant guidelines and regulations in the Third Hospital of Hebei Medical University. This study was approved by the ethics committee of the Third Hospital of Hebei Medical University (No. Ke2014-004-1) and was conducted in accordance with the Helsinki Declaration. Patients were contacted by telephone to ensure that they agreed on participating in the study, and informed consent was obtained from all participants or their legal guardians. OA was diagnosed by clinicians according to the American College of Rheumatology criteria [6].

The inclusion criteria were OA patients with moderate to severe symptoms, and a Kellgren–Lawrence (KL) score of >2 points, or a knee joint with varus deformity and medial space narrowing. The exclusion criteria were post-traumatic knee OA, inflammatory joint disease, or a history of previous operations or fractures. Patients who fitted the inclusion criteria, but refused the treatment, and those who had radiographic evidence of significant varus of lower extremity were also excluded in our research.

5 patients abandoned the research program for personal reasons or lost to follow-up. The individual pictured in our research has provided written informed consent (as outlined in PLOS consent form) to publish their image alongside the manuscript. Totally 49 subjects or 53 knee were evaluated (two weeks before or after one year) with a minimum of one year postoperatively. Among them, 13 knees of males and 40 knees of females were included. The average age of patients was 67.1 ± 6.82 years (range from 51 to 87 years) and the duration of disease was 6.14 ± 3.31 years (range from 1.3 to 22 years). 53 limb were classified as Knee OA grade 3 (35), grade 4 (18).

All clinical and radiological data were prospectively collected. The data, including demographics, pre-operative clinical and radiological weight bearing whole lower extremity radiographs, and post-operative data were analyzed after a minimum of one year follow up post-operatively. The authors had not access to information that could identify individual participants during or after data collection.

### Surgical technique

The surgical procedure had been described in previous study [1]. Firstly, an incision about 3 to 5 cm that located at the proximal of the fibula was made. The fascia was then opened along with the septum between the peroneus and soleus, then the muscles were separated, and fibula was exposed. A 2-cm width of the fibula was removed at 6 to 10 cm below the fibular head with the use of an oscillating saw. The fibular fracture ends were covered with bone wax after the resection. The muscles, fascia, and skin were then sutured separately. All the operation was conducted by the same surgeon (Y Zhang) for every patient of the study. Postoperatively, the patients were allowed to ambulate as soon as possible.

### Radiographic evaluation

**Fig 1:**
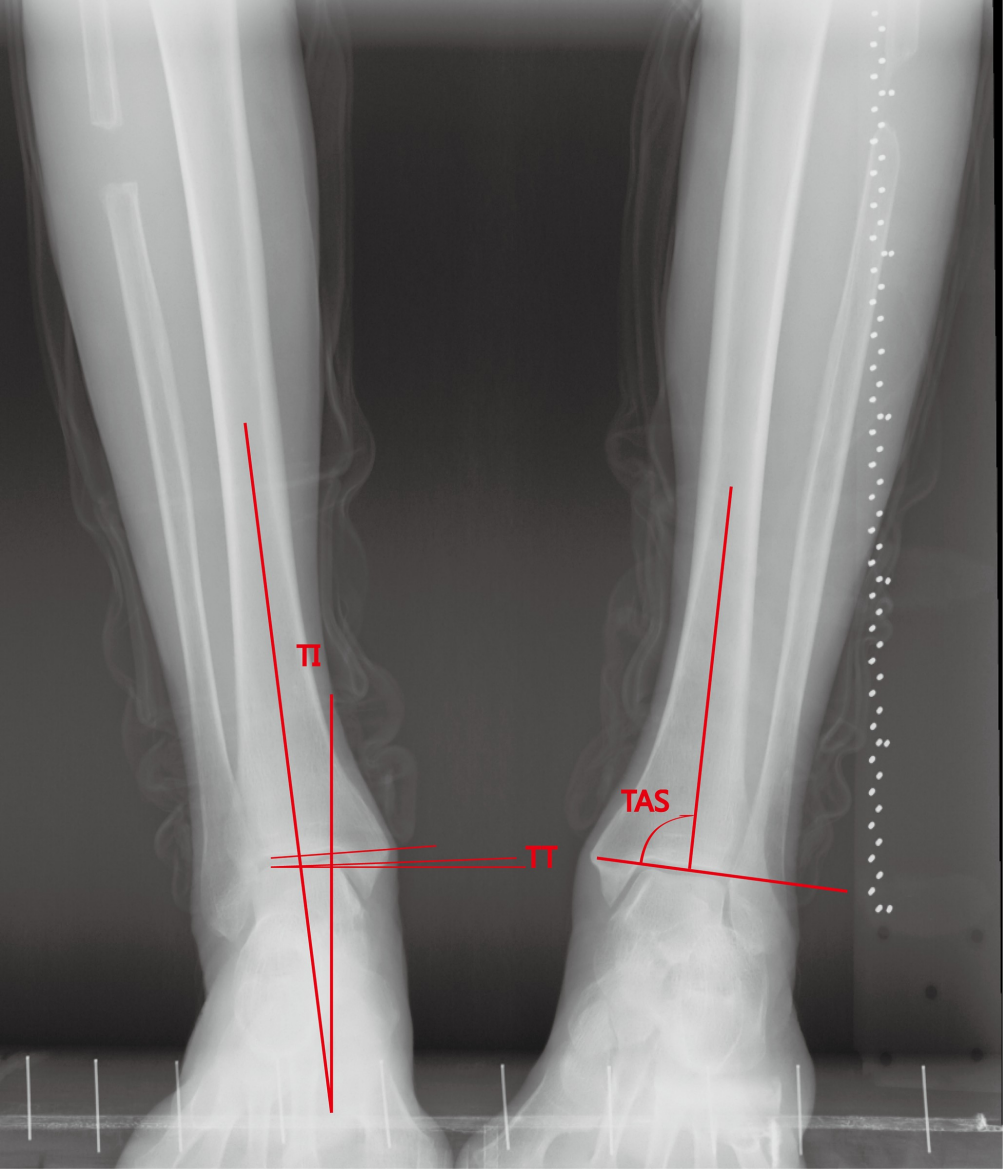
Definition of the different angles from antero-posterior radiographs. Tibial inclination (TI): acute angle between the perpendicular line and the tibial axis. Talar tilt (TT): acute angle between the tangent of the horizontal line and the superior talar surface. Distal tibial articular surface (TAS): the angle between the distal tibial joint surface and the tibial shaft.

**Fig 2:**
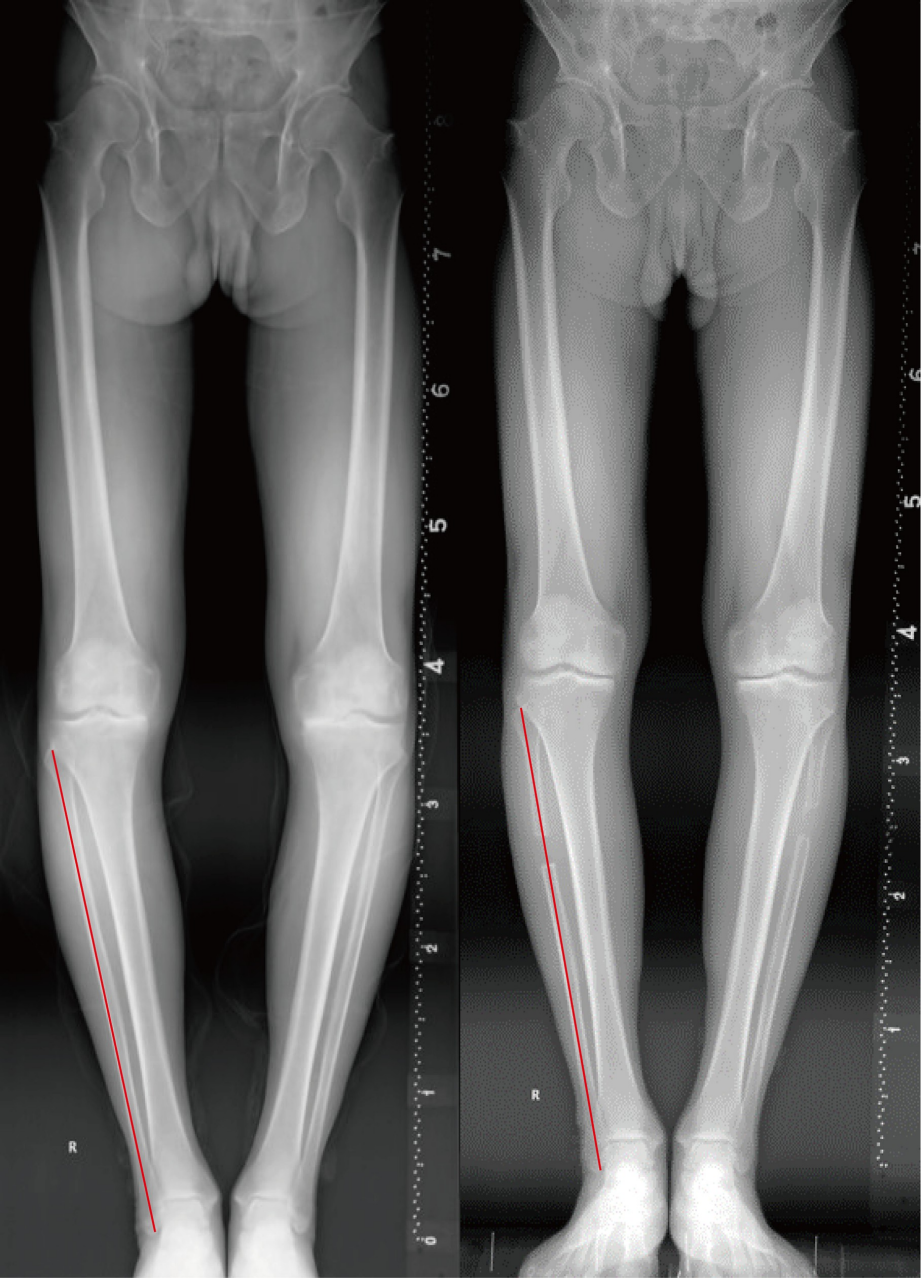
The measurement of proximal migration of lateral malleolar. The fibular length was measured. To minimize the error, the proximal point was fixed at the intersection of fibular and tibial cortical, the distal point was located at the tip of lateral malleolar.

**Fig 3:**
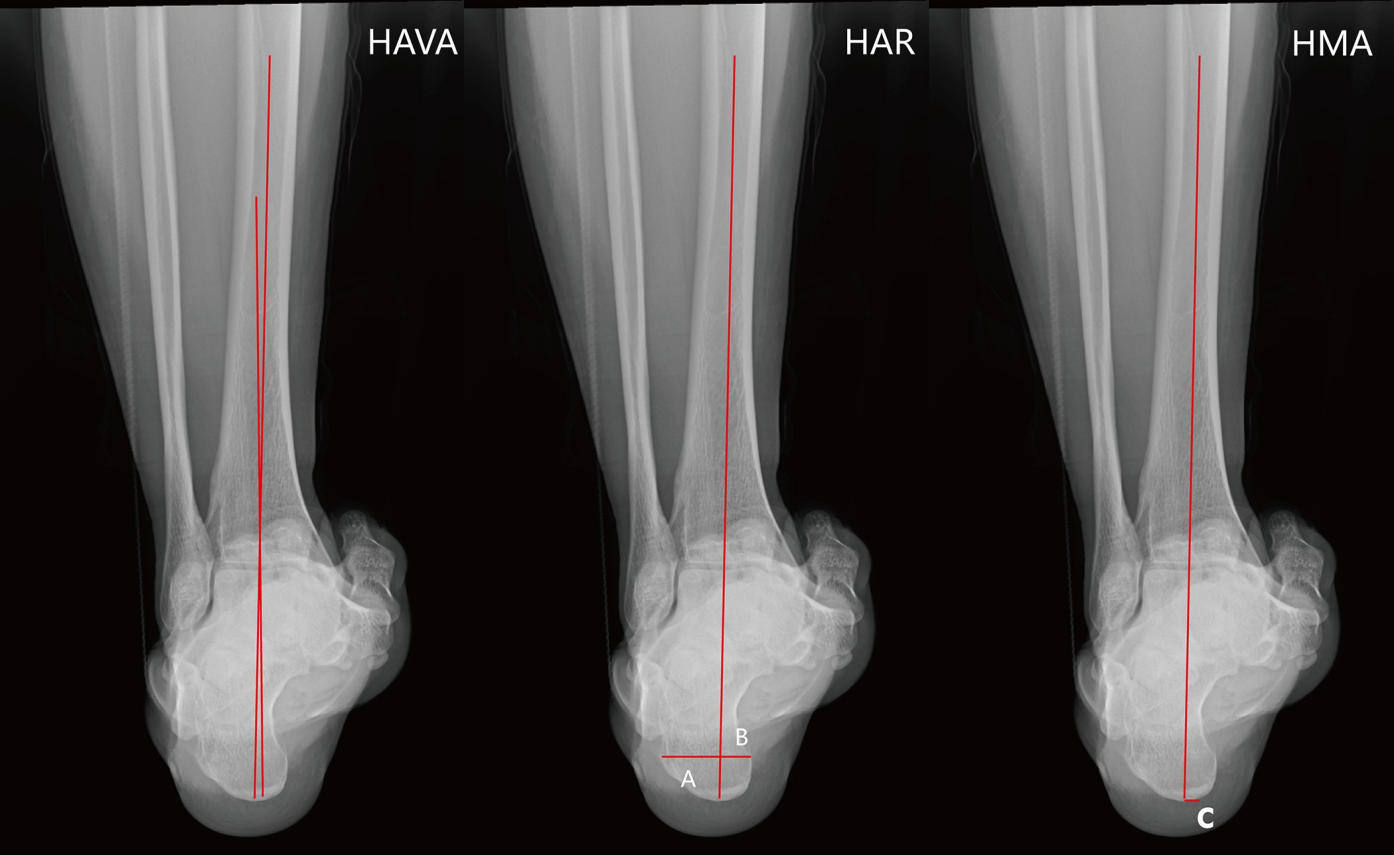
Measurements of hindfoot alignment. The angle between a line drawn from the most inferior point of calcaneus to joint line the intersection and the tibial axis was defined as the hindfoot alignment view angle (HAVA). The hindfoot alignment ratio (HAR) was calculated as B/A, the widest point width of the calcaneus was divided as medial and lateral part by the tibial axis (B and A). The vertical distance from the most distal point of calcaneus to the tibial axis was defined as the hindfoot moment arm (HMA) (C).

Pre- and post-operative standing ankle and tibial AP views with one-leg weight bearing were taken to assess the hip-knee-ankle angle (HKA), the femoro-tibial angle (FTA: the lateral angle between the femoral anatomical axis and the tibial anatomical axis) [7], tibial inclination (the acute angle between the perpendicular line and the tibial axis, TI), distal tibial articural surface (the angle between the distal tibial joint surface and the tibial shaft and, TAS), talar tilt (the acute angle between the horizontal line and the tangent of superior talar articular surface, TT) (**Fig** 1). The proximal migration of lateral malleolar was also monitored by measuring the length of fibular in PACS. To minimize measuring error, the proximal point was fixed at the intersection of fibular and tibial cortical, and the distal point was located at the tip of lateral malleolar (**Fig** 2). The hindfoot alignment view angle (HAVA), hindfoot alignment ratio (HAR), and hindfoot moment arm (HMA) were measured on hindfoot alignment radiographs with method that were described by other reseachers [8, 9]. The angle between a line drawn from the most inferior point of calcaneus to joint line the intersection and the tibial axis was defined as the hindfoot alignment view angle (HAVA). The hindfoot alignment ratio (HAR) was calculated as B/A, the widest point width of the calcaneus was divided as medial and lateral part by the tibial axis (B and A). The horizontal distance from the most distal point of calcaneus to the tibial axis was defined as the hindfoot moment arm (HMA) (C). (**Fig** 3). The assessment were repeated after two weeks. The intra-class correlation coefficient (ICC) was used for evaluation of the intra-and inter-observer reliabilities which exhibited a good reliability (ICC>0.9).

### Clinical evaluation

Before surgery and at the last post-operative outpatient visits, a standardized clinical exam was conducted and assessed with the questionnaires of the American Knee Society Score (KSS), the Ankle-Hindfoot Scale of the American Orthopedic Foot and Ankle Society (AOFAS), and Visual Analogue Scale/Score (VAS) which were commonly used methods of assessment for ankle functions.

### Statistical analysis

The Kolmogorov Smirnov test was carried out to test the normality of the distributions. The continuous variables were described as the mean values and standard deviation (SD). Frequencies were used to express categorical data. The statistical analysis was performed with SPSS 21.0 (SPSS Inc., Chicago, IL, USA). The Pearson chisquare test and the nonparametric Mann–Whitney U test were used to identify significant differences between two groups. < 0.05 represented a statistically significant difference.

## Results

To address potential sources of bias and potential confounders, all operations were performed by one senior orthopedic surgeon (corresponding author) in our department, all the classification were diagnosed by first author and reviewed by corresponding author twice to ensure the accuracy of data in our article. The study size was larger than 30, and it was enough to conclude satisfying results. And the average time between injury and surgical intervention was 2 days (1–4 days) which mainly due to delay in reporting to the hospital or time taken to examine patients’ biochemical indices (S1 Table).

### Radiographic evaluations

**Fig 4:**
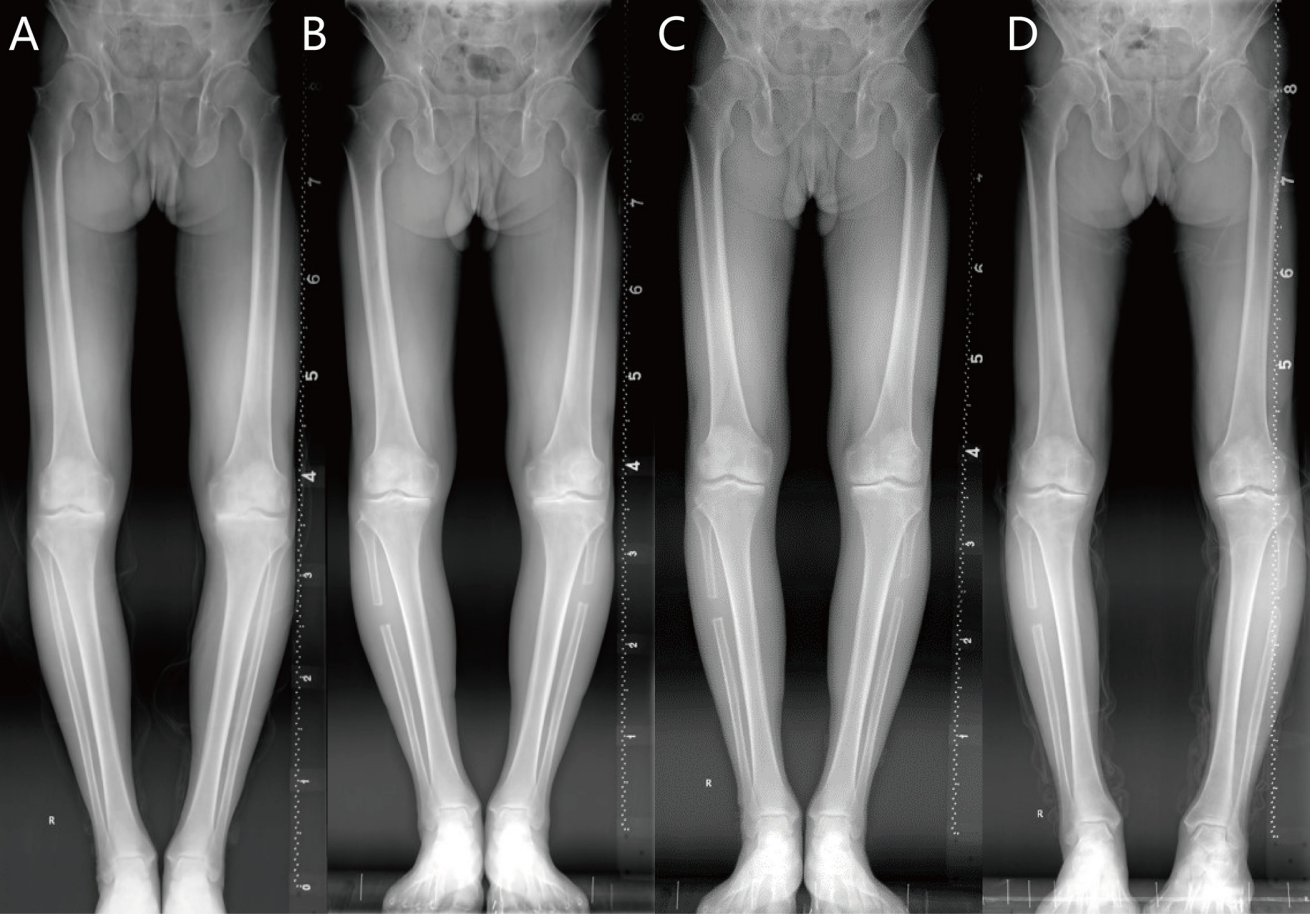
Preoperative and postoperative radiographs of a 68-year-old man. (A) Preoperative radiographs exhibited that a severe degree of medial compartment osteoarthritis. (B) Postoperative radiographs after PFO immediately, the alignment of knee and ankle was not changed obviously (C) Postoperative radiographs at 3 months after PFO shows that the recovered medial joint space and improved alignment of ankle (D) Postoperative radiographs at 1 years after PFO shows that medial joint space and improved alignment of ankle was maintained with some decrease.

The HKA was corrected from 171.0 ± 3.3° to 173.0 ± 2.8° (*P*<0.001). The mean FTA was improved from a preoperative value of 185.0 ± 1.9° to 180.5 ± 1.3° after follow-up (*P*<0.001). The TI was corrected from 7.5 ± 2.0° to 6.6 ± 1.8° (*P* = 0.019), and the TT was improved from 11.9 ± 2.0° to 7.1 ± 1.6° (*P*<0.001). The mean TAS angle was 80 ± 3.5° that was not changed before and after surgery (Table 1). Prior to PFO, the axis of tibia was inclined laterally, meanwhile the distal tibial joint surface inclined to the varus, and the talus inclined to medially. After PFO, the tibial lateral inclination decreased along with the distal tibial joint surface inclines to the valgus (although most of patients were neutral or still varus), and the medial inclination of the talus was also decreased. These effects lead to an improvement in tibio-talat joint adaptation (**Fig** 4). As hindfoot alignment, the 12-month postoperative HAVA, HAR and HMA had significantly increased after PFO (P < 0.001), however valgus deviation of the hindfoot were still existed. The length of fibular was decreased, but there were no significant differences which means that the proximal migration of lateral malleolar was not obvious.

**Table 1:** Preoperative and final follow-up radiological measurement.

	Preoperative	Final Follow-up	P Value
HKA, degrees	171 ± 3.3°	173 ± 2.8°	<0.001
PTA, degrees	185 s 1.9°	180 .5± 1.3°	<0.001
TAS, degrees	80 ± 3.5°	80 ± 3.5°	-
TT, degrees	11.9 ± 2.0°	7.1 ± 1.6°	<0.001
TI, degrees	7.5 ± 2.0°	6.6 ± 1.8°	0.019
FL, cm	36.1 ± 1.8	35.9 ± 1.9	0.66
HAVA, degrees	-5.8 ± 3.6°	-3.4 ± 2.5°	<0.001
HAR	0.1 ± 0.2	0.2 ± 0.1	<0.001
HMA, mm	-11 ± 6.1	-8.2 ± 4.7	0.045

### Clinical evaluation

**Fig 5:**
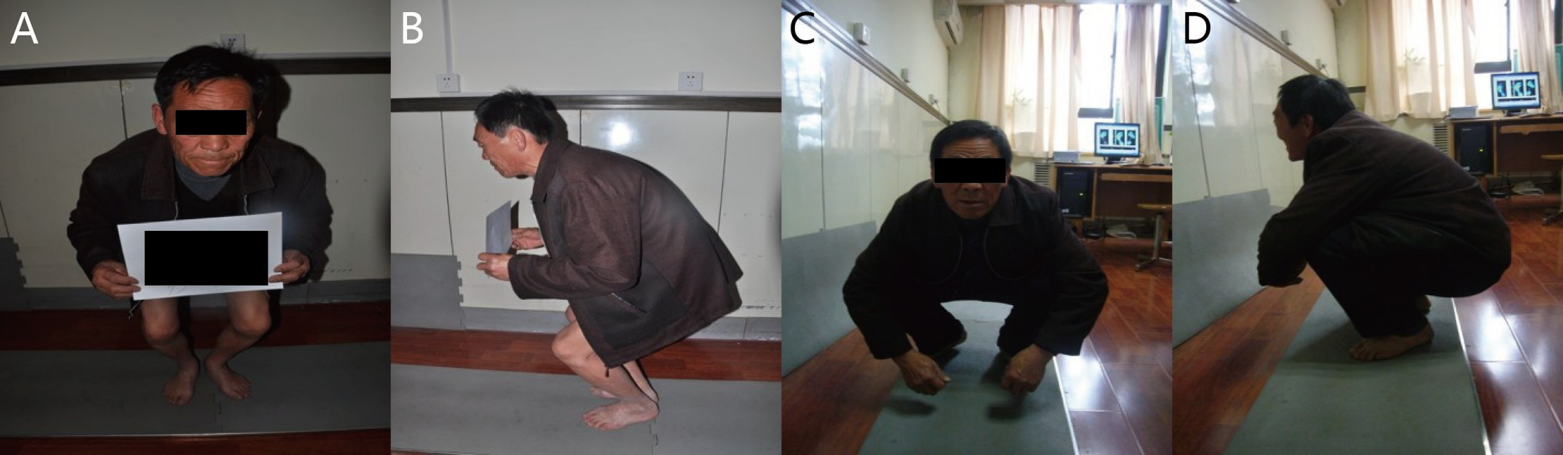
Preoperative and postoperative clinical assessment of the 68-year-old man. (A) (B) Preoperative photographs exhibited that a limited range of motion. (C) (D) Postoperative photographs at 1 years after PFO, the improved range of motion in knee joint.

The alignment of knees was corrected partly from varus in all affected legs of 53 subjects with no complications. The average preoperational KSS, AOFAS and VAS score were 45.4±10.3 points (range from 25 to 55 points), 89.9±3.98 points (range from 79 to 95 points) and 6.9±1.0 points (range from 5 to 8 points), respectively. After PFO, a significant difference was exhibited in KSS (63.5±10.8, range from 45 to 82 points), but no differences was illustrated in AOFAS (91.1±3.7, range from 85 to 95 points) and VAS scores (7.2±1.2, range from 5 to 9 points) (Table 2) (Fig 5).

**Table 2:** Preoperative and final follow-up clinical measurement.

	Preoperative	Final Follow-up	P Value
KSS, scores	45.4±10.3	63.5±10.8	<0.001
AOFAS, scores	89.9±3.98	91.1±3.7	0.123
VAS, scores	6.9±1.0	7.2±1.2	0.134

## Discussion

Varus deformity results in medial compartment compression, the narrowing of joint space and weight axis shift in medial compartment KOA patients. PFO is a procedure proposed by us to treat medial compartmental osteoarthritis of knee. It becomes an alternative treatment for osteoarthritis of the medial compartment, especially for those who cannot undergo TKA because of their medical comorbidities. There have been numerous clinical and experimental studies focusing on the angular deformity changes of the knee joint after PFO, but reports about effects on ankle joint are not found in literature [5,10]. The research focuses on ankle alignment changes after PFO, and find that the alignment of ankle joint is improved significantly. This study enriches our understanding of PFO in treating medial compartment osteoarthritis, and provides data to support this technique in treating knee diseases.

The fibular is an important structure in maintaining the balance of the periarticular soft tissue and supports one-sixth of body weight [11]. The PFO can be explained by the theory of non-uniform-settlement in the treatment of medial compartment KOA [5]. After PFO, the proximal fibula is no longer subject to constraints from distal fibula, and the tensile force is then transmitted to lateral femoral condyle. Therefore, the knee varus deformities resulting from load bearing is reduced, and the pressure on the medial compartment is also decreased. The patients’ medial knee pain is then relieved obviously. Consistent with the theory, a significant differences in PTA and HKA were exhibited in our research, and the varus deformity was reduced significantly after PFO. Besides, the PFO will not interfere the procedure of TKA in the following necessary revision.

There have been researches focusing on the correlation of ankle and knee joints after the treatment with HTO, but no consensus are concluded. Takeuchi reported the successful treatment to osteoarthritis of ipsilateral knee and ankle joints with HTO [11]. They found that the tilt of ankle joint as well as the varus deformity of knee joint was improved after HTO, and suggested HTO was one of the effective methods in treating medial compartment osteoarthritis of knee and ankle on the same leg. On the contrary, Jeong reported that the varus deformity can be corrected by HTO, but the valgus alignment in ankles should be treated by closing wedge medial supramalleolar osteotomy in some patients [12]. Similar to HTO, PFO is considered as an effective surgical procedure for patients with varus deformity and medial compartment osteoarthritis, but the effect on ankle after the changed limb alignment was not fully studied. With a cadaver study, Balditi reported that a decreases in peak force, peak contact area, and peak pressure at all flexion angles were exhibited after PFO in the tibiotalar joint [13]. However, they did not identify stress distribution in different part of ankle joint, and true gait cannot be simulated accurately in cadaver research. In our research, the deformity in ankle was indeed corrected after PFO, but functional evaluation of ankle assessing by AOFAS and VAS scores were not significantly changed. One reason is that the functional changes of ankle may be covered up by severe symptom in knee after PFO. The other reason to the negative functional results can be explained that the patients enrolled in our research are diagnosed with medial compartment KOA, but not with concurrent spontaneous osteoarthritis of the ipsilateral ankle. It is possible for them to have unaltered results, and only patients with ipsilateral osteoarthritic ankles are more likely to achieve significant improvements in clinical symptoms.

Different from functional evaluation, the improvement was significant in radiographic evaluation after PFO. The tilt of ankle joint (TI) which reflects the position of tibia is improved, and significant differences are also exhibited in TT which means the alignment of ankle joint is corrected simultaneously. Improvements in HKA and FTA reflecting limb alignment changes is also exhibited, and the varus deformity is reduced. The changes in alignment are bound to lead changes in stress distribution. In fact, the overall loading on ankle joint after PFO is unchanged because of the entire load transmitting from the intact tibia to ankle joint was still same. But the surgery to fibular inevitably results in redistribution of load in ankle joint after PFO. Balditi considered that partial fibular osteotomy made weight bearing to move to far medial aspect of tibiotalar joint [13]. Due to experiment limits, the changes cannot be recorded in our research. Furthermore, the lateral malleolar was not significant immigrated proximally, and this is benefited to sustain the stability of ankle joint. Degenerated disease is considered as part of the normal aging process and varies in joints using as load bearings such as knees, ankles, and the process might be accelerated when the deformity or abnormal alignment of limb exists. Although no significant differences are concluded in clinical evaluation, the significant structural improvement of ankle joint is still benefit to the correction of alignments and relieves of ankle symptoms partly in patients at the end of follow up.

Although PFO does not require insertion of additional implants and exhibits excellent effect on medial compartment KOA patients, the effect on varus deformity of tibial plateau and alignment of ankle are limited. We notice that the function of ankle is improved but with limited extent in radiographic results. Secondly, although anatomical varus angulation is corrected partly, none of these patients have an anatomic valgus alignment postoperatively. Meanwhile, preoperative hindfoot valgus deviation was significantly decreased but deformity was still existed after PFO. Thirdly, no significant differences were demonstrated in clinical evaluations of ankle (AOFAS and VAS scores). In our previous study, medial joint space width is also considered as an independent factors affecting postoperative clinical outcome after PFO [5]. Therefore, the PFO has its own limitations in correcting alignment of lower limb. For these reasons, we propose that PFO is suitable to treat patients with varus deformity smaller than 5 degree in knee joint. For severe ones with varus deformity larger than 5 degree, restorable spacers and four point support plates have been created and used to correct the alignment of lower limb in clinic, and exhibits excellent results. Compared with patients treated with HTO or arthroplasty, the advantage of PFO and new devices are that they delays time of involvement to another bone (tibia), and make it possible to provide more options for those patients who need revised operations. In following research, the patients treated by PFO or PFO combined with restorable spacer will be compared and concluded.

There are some limitations in our research. The process of measuring angle could have been influenced. However, the angle was measured by independent observers repeatedly, who showed a high reproducibility of 0.94. Secondly, a control group is better to compare the differences, but a placebo control is difficult to include when performing this surgery because of the inability to exclude a placebo effect.

In conclusion, after PFO, the alignment of ankle is improved obviously. Although there is not a direct correlation between the structural improvements with clinical function, PFO not only improves function in knee, but also correct the alignment of ankle joint, and is still recommended as a safe surgery treating medial compartment osteoarthritis. In following research, patients with medial compartment KOA combined with ankle arthritis will be studied to identify the advantages of PFO on ankle joint.

## Supporting information

S1 Table The original data of our research about measuring. (XLSX)
